# Chicago Medical Society

**Published:** 1867-01

**Authors:** 


### CHICAGO MEDICAL SOCIETY.

At the meeting of this Society on the evening of the 21st of December, 1866, Dr. Ira Hatch related a fatal case of cerebrospinal meningitis, or spotted fever, that had occurred in his practice recently. During the discussion that followed the relation of the case, Dr. Orrin Smith referred to the efficacy of sulphuric acid in the treatment of erysipelas and small-pox, claiming that when given in efficient doses, its effects were more promptly curative than the tincture of chloride of iron.

At the same meeting of the Society, Dr. Quales presented the uterus of a female recently dead from puerperal or childbed fever, in the County Hospital. It showed the usual appearances of severe inflammation.

At the meeting on the evening of Dec. 28th, 1866, the question for discussion was as follows:—"In what class of cases, and when, are surgical operations justifiable, in the treatment of cancerous diseases?"

Dr. Bogue, in opening the discussion, remarked that, as a general rule, the results of operative procedures for the removal of cancerous tumors were unsatisfactory. Epithelial cancers and scirrhus in its early stage, are the varieties most likely to be permanently benefited by extirpation. As a general or average result, thinks it doubtful whether life is prolonged by surgical interference. Regards operations sometimes justifiable for the purpose of giving the patient temporary relief from the great pain and offensiveness of the encephaloid cancerous growths in their advanced stage. When the disease affects one of the extremities, he regards amputation as preferable to the simple extirpation of the tumor.

Dr. G. Paoli said he thought the profession divided in opinion, as to the value of surgical operations in cancerous diseases. Statistics appear to show that a large majority of cancerous patients die in from 12 to 18 months after surgical operations for their relief. He claimed that many cases of the epithelial variety of cancer, and some cases of scirrhus, were benefited by operations for their removal. But if the latter variety existed in both breasts, or involved the neighboring glands, or the patient had attained an age beyond 55 or 60 years, no operative procedures should be undertaken.

Dr. Nelson related an interesting case of supposed cancerous tumor in the axilla of a soldier, which was extirpated in Boston, in 1862 or 1863, and up to this time the man remains well.

Dr. E. L. Holmes remarked that the case related by Dr. Nelson, and others like it, certainly justified operations to some extent. He also thought an operation sometimes justifiable, simply to obtain temporary relief from severe suffering and annoyance. He thought, however, that the diagnosis of cancerous tumors was in some cases extremely difficult; the results of microscopic examinations not affording a sufficient test.

The discussion was further continued by Drs. N. S. Davis and H. M. Lyman, both of whom advocated the propriety and importance of paying more attention to the constitutional treatment of cancerous diseases.

ELGIN MILK CONDENSING COMPANY.

Dr. Hamill reported the observations made by himself and Dr. H. A. Johnson, during a visit to the "Elgin Milk Condensing Company," which were of a very satisfactory character. The whole process, from the delivery of the milk by the dairymen, until it was prepared in its condensed form to be delivered to consumers, was exhibited to them. The integrity of the parties, the scrupulous care with which the milk is received, the cleanliness and sweetness of every vessel through which it passes, are guarantees to the public for its good qualities. It is recommended to the profession and the community for the following reasons:—

1st. It is procured from healthy cows, that are not fed with slops from the distilleries, and are not kept, during any part of the year, in dark, close, and dirty barns.

2d. The process in no way deteriorates the quality of the milk, and its bulk is diminished, making its transportation easy.

3d. It is of uniform strength, requiring three parts of pure water to one part of condensed milk to bring it to the standard of good fresh milk. There is no drug or chemical used or mixed with it.

And, lastly, it can be kept for a much longer time, at any season of the year, from becoming sour, if proper care is taken.

It is the pecuniary interest of the company to use good milk for condensing, and none other; this furnishes an additional guard over the purity of the article.

In consideration of these facts, it is especially adapted to the nourishment of infants who are deprived of a mother's care, or of good milk; if it supplies no other want, it will confer an unlimited blessing on this dependent class of human beings.

The following letter from Prof. Johnson to Dr. H., will be read with interest by all, and needs no comment:—

Dr. Hamill—*Dear Sir:*—I received from Mr. Hinckley a specimen of the milk, condensed at Elgin in our presence, and have subjected it to an examination, for the purpose of ascertaining what changes have taken place in its constitution. I added to one part of the "condensed milk" three parts of water, mixing well. The sp. grav. was then 1030, water being 1000. The casein did not differ, so far as I could judge, from that of good uncondensed milk. The amount of cream was

*within* the range of good new milk. The corpuscles, as seen under the microscope, to a *very limited* extent, were broken, and in size, were somewhat more variable than those of good uncondensed milk, the effect, I presume, of the mechanical agitation during the process of condensation. The taste of the milk, after condensation and dilution, as compared with specimens of the same milk before condensation, was somewhat richer, giving the impression of more cream, due, I think, to the rupture, as previously stated, of the envelopes of some of the corpuscles. This, while it gives a richer taste, in no way impairs the quality of the milk. I used in my examination, as a standard of comparison, the new milk from my own cow, a healthy animal, yielding, as we think, milk of an excellent quality. This process in no way injures the quality of the milk, while it diminishes the bulk for transportation; is clean; is not likely to be adulterated, and may be kept without change for a much greater length of time. I take pleasure in saying that, in my judgment, the public may rely upon it implicitly, and I trust that the gentlemen interested may find sufficient encouragement to induce them to continue its preparation.

In conclusion, I beg leave to suggest, that, as in all milk adulterated, whether with water alone, or with water and an admixture of other ingredients, the cream is invariably diminished in relation to its bulk or quality, that the galactometer may be advantageously resorted to as a means of examination in cases of suspected falsification. This instrument consists of two glass tubes, of considerable size, connected by a smaller tube. This latter has a graduated scale attached. The instrument is filled with milk up to the small tube, a portion of acetic acid is added; the two well shaken together for a minute or two, when the butter, liberated by the acid, rises to the surface and fills the smaller tube. If gently warmed it will become limpid, and the amount will be indicated by the graduation of the smaller tube. If it be desirable to determine the proportion of cream to the whole amount of milk, the capacity of the instrument and the value of the divisions on the scale should be determined. If, for instance, the capacity of the larger tube be five times that of the smaller, and the tube be divided into ten equal parts, it would be easy to determine, within a very small fraction, the relative proportions of cream in the milk with which the instrument is charged.

This process does not give, it is true, a scientific analysis of milk, but indicating, as it does, the amount of cream, its results are sufficient for all practical purposes.

Very truly, yours,

H. A. Johnson.

611 Wabash Avenue, December 28th, 1866.

After hearing the report of Dr. Hamill, the following resolution was offered by Dr. N. S. Davis, and unanimously adopted:

*Resolved*, That the great importance of having milk pure, uniform in quality, and capable of remaining sweet longer than that ordinarily distributed directly from the dairy, makes the condensed milk furnished by the Elgin Milk Condensing Company an article of great value to the community, and one which we freely recommend for general use, and especially for use in feeding children.

